# Characterization of a Natural Mutator Variant of Human DNA Polymerase λ which Promotes Chromosomal Instability by Compromising NHEJ

**DOI:** 10.1371/journal.pone.0007290

**Published:** 2009-10-06

**Authors:** Gloria Terrados, Jean-Pascal Capp, Yvan Canitrot, Miguel García-Díaz, Katarzyna Bebenek, Tomas Kirchhoff, Alberto Villanueva, François Boudsocq, Valérie Bergoglio, Christophe Cazaux, Thomas A. Kunkel, Jean-Sébastien Hoffmann, Luis Blanco

**Affiliations:** 1 Centro de Biología Molecular Severo Ochoa (CSIC-UAM), Madrid, Spain; 2 Laboratoire d'Ingénierie des Sistèmes Biologiques et des Procédés. UMR INSA/CNRS, Toulouse, France; 3 CNRS-IPBS (Institute of Pharmacology and Structural Biology), Toulouse, France; 4 Laboratory of Molecular Genetics, National Institute of Environmental Health Sciences, Research Triangle Park, North Carolina, United States of America; 5 Clinical Genetics Service, Department of Medicine, Memorial Sloan-Kettering Cancer Center, New York, New York, United States of America; 6 Laboratory of Translational Research, Institut Català d'Oncologia-IDIBELL, L'Hospitalet de Llobregat, Barcelona, Spain; University of Minnesota, United States of America

## Abstract

**Background:**

DNA polymerase lambda (Polλ) is a DNA repair polymerase, which likely plays a role in base excision repair (BER) and in non-homologous end joining (NHEJ) of DNA double-strand breaks (DSB).

**Principal Findings:**

Here, we described a novel natural allelic variant of human Polλ (hPolλ) characterized by a single nucleotide polymorphism (SNP), C/T variation in the first base of codon 438, resulting in the amino acid change Arg to Trp. *In vitro* enzyme activity assays of the purified W438 Polλ variant revealed that it retained both DNA polymerization and deoxyribose phosphate (dRP) lyase activities, but had reduced base substitution fidelity. Ectopic expression of the W438 hPolλ variant in mammalian cells increases mutation frequency, affects the DSB repair NHEJ pathway, and generates chromosome aberrations. All these phenotypes are dependent upon the catalytic activity of the W438 hPolλ.

**Conclusions:**

The expression of a cancer-related natural variant of one specialized DNA polymerase can be associated to generic instability at the cromosomal level, probably due a defective NHEJ. These results establish that chromosomal aberrations can result from mutations in specialized DNA repair polymerases.

## Introduction

The maintenance of genome integrity is dependent on numerous mechanisms, which notably allow fidelity of DNA replication and repair of damaged DNA [1]. Those processes require a large number of proteins including DNA polymerases. Nevertheless, the recent discovery that eukaryotic cells contain many more DNA polymerases than previously thought added further complexity to our appreciation of DNA transactions (review in [2]). Function of those recently discovered DNA polymerases remain still uncertain but numerous connections between their regulation, organisation, and coordinated action for DNA protection have been already made [3]. A novel family X DNA polymerase, named Polλ, has been independently identified in three different laboratories [4]–[6]. Polλ forms a Polβ-like core that consists of two domains: 31 kDa polymerization domain (bearing the three conserved subdomains: fingers, palm, thumb) and 8 kDa domain [7]. In agreement with their structural relationships (32% amino acid identity), the biochemical properties of Polλ are partly similar to those of Polβ, and suggest a role in DNA repair [8]. Indeed, as Polβ, Polλ has a dRP lyase activity [9], and accordingly, these enzymes both have a role in BER [10]–[13]. However, unlike Polβ, Polλ contains a BRCA1 C-terminal (BRCT) domain [4], [14], [15], required for a stable interaction with NHEJ factors [16]–[18]. Moreover, Polλ is able to perform alignment-based gap filling for NHEJ in human nuclear extracts [19], and the expression in mammalian cells of a catalytically inactive form of Polλ decreases the frequency of NHEJ events in response to I-Sce-I –induced DSB [20]. All these features support a potential role for Polλ in the NHEJ repair of DSB.

A number of polymorphic variants have been described in several DNA repair genes that could -when adequately combined- substantially alter overall DNA repair capacity. Conversely, few reports exist on the identification and characterization of polymorphic or altered isoforms of the known DNA polymerases, with the exception of Polβ [21]–[24]. Here, we report the identification of a natural allelic variant of hPolλ that has reduced base substitution fidelity *in vitro* and whose expression in cultured cells increases mutation frequency and compromises the DSB repair pathway NHEJ, resulting in radiosensitivity and chromosomal instability.

## Results

### Identification of a SNP in the coding region of hPolλ

**Figure 1:**
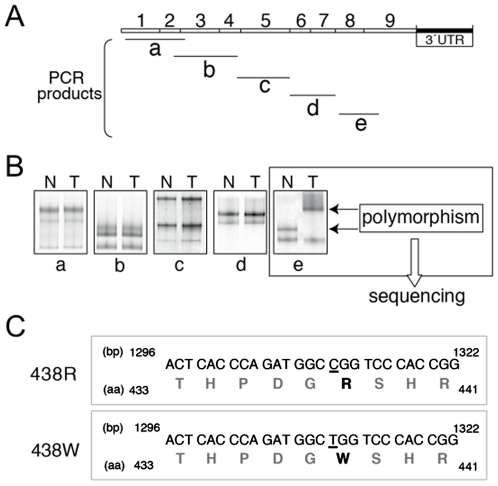
PCR/SSCP analysis of the coding region of hPolλ gene. (A) Five overlapping fragments, named a–e, covering the whole coding region of the gene were amplified as described in Materials and Methods. (B) SSCP analysis of fragments a–e from non-paired normal (N) and ovarian tumor (T) samples. Only fragment e showed an altered mobility pattern (indicated by arrows). (C) Sequencing of fragment e identified a single nucleotide change (C->T) at nucleotide position 1311 of the cDNA sequence that changes the wild-type arginine residue (R438) to a tryptophane (W438).

Several normal and tumoral cDNA samples were screened for possible nucleotide changes in the hPolλ gene. The complete coding region of hPolλ, comprising exons 1 to 9, was amplified by polymerase chain reaction (PCR) in five overlapping fragments, named a–e (Fig. 1A), and subjected to single-stranded conformational polymorphism (SSCP) analysis. Figure 1B illustrates a representative analysis using non-paired normal and tumor ovarian tissues. As shown, fragments a, b, c and d, covering the first seven exons of hPolλ, did not produce any band with altered mobility when normal and tumoral samples were compared. Moreover, no mobility alteration was detected for fragments a–d in any other tissues analyzed. However, PCR-SCCP analysis of fragment e (covering exon 8 and the first half of exon 9 of the transcript) identified an abnormal mobility pattern when comparing normal ovary *vs* ovarian carcinoma GI-101 (Fig. 1B). The same variation in the PCR-SSCP profile for fragment e was identified using other tissues from the normal and tumoral panel.

Sequence analysis of the PCR fragment e, revealed the existence of a SNP, C to T variation in nucleotide position 1311 (exon 8) of the coding region of hPolλ, resulting in a single arginine (R) to tryptophan (W) amino acid substitution in codon 438 (Fig. 1C). SNP reported here has been designated rs3730477 (http://www.ncbi.nlm.nih.gov/SNP/snp_ref.cgi?rs=3730477), and is one of the two SNPs altering the coding sequence identified to date in hPolλ.

### hPolλ W438 variant has normal polymerase and dRP lyase activities

**Figure 2:**
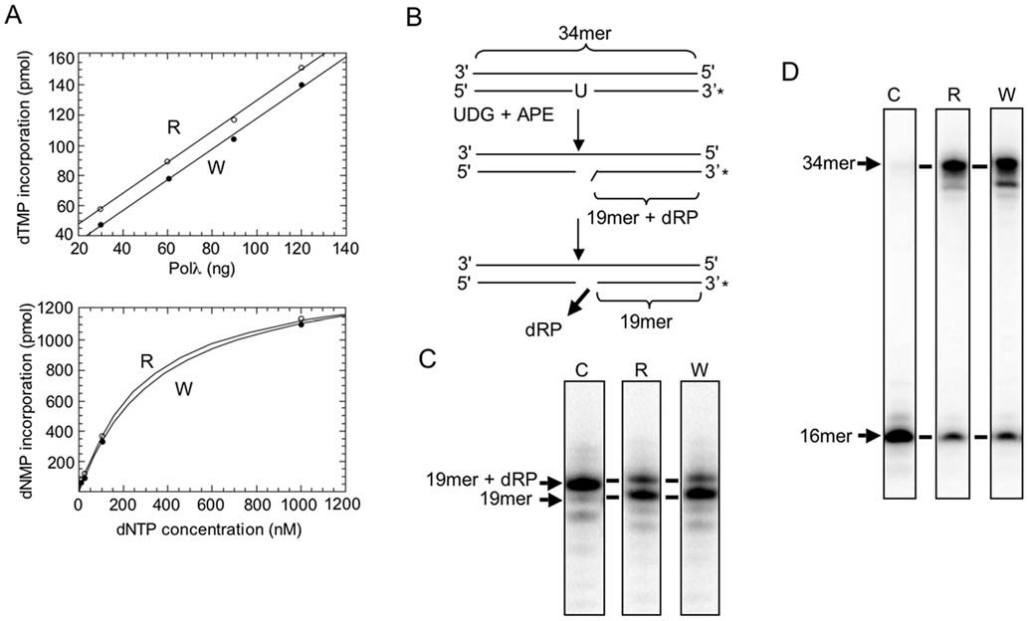
Enzymatic activities of the hPolλ W438 variant. (A) DNA polymerization activity of the two allelic variants of hPolλ. DNA polymerase activity of each independent hPolλ variant (R438 or W438), measured as dNMP incorporation on activated DNA, was estimated as a function of enzyme (top) and dNTP (bottom) concentration, as described in Materials and Methods. The first experiment, in which the only dNTP provided was TTP (13,3 nM), demonstrates that nucleotide insertion was linear for both variants, in the interval of 30–120 ng of enzyme. The second experiment, using 60 ng of each enzyme variant, tested polymerization of the four dNTPs at various (from limiting to saturating) concentrations. (B–D) dRP lyase activity and reconstitution of BER *in vitro* with the two allelic variants of hPolλ: (B) The scheme shows a 34-mer double-stranded oligonucleotide containing an uracil residue (at position 16) in the strand which is 3′-end labeled (*). After treatment with UDG and hAPE, a dRPcontaining nicked substrate (19mer+dRP) is obtained, that can be a substrate for dRP lyase activity. (C) *In vitro* analysis of the dRP lyase reaction. As shown in the autoradiogram, the dRP moiety can be cleaved by incubation with either variant (R or W) of hPolλ (30 nM). (D) *In vitro* reconstitution of a BER reaction. A non-labeled 34-mer double-stranded oligonucleotide containing a uracil residue at position 16 in one strand is treated with UDG (100 nM) and hAPE (40 nM) to release a dRP-containing nicked substrate. By adding a labeled dNTP (α-dCTP) and either purified hPolλ R or hPolλ W variants (60 nM), two labeled products can be observed after denaturing electrophoresis and autoradiography: (i) a 16-mer product generated by a single nucleotide insertion at the 3-hydroxyl end of the 5-incised AP site; (ii) a 34-mer product that corresponds to the complete repair of the DNA strand upon T4 DNA ligase action.

Polλ has been proposed to be a suitable candidate to participate in BER [12], [13], as it contains both gap-filling DNA synthesis and dRP lyase activities [8], [9]. To address if the W438 mutation affects any of these activities, the wild-type form (R438) and the W438 allelic variant were overexpressed in *E. coli* cells and purified in parallel to compare their biochemical properties. Both enzymes showed a similar DNA polymerization capacity, and a similar affinity for dNTPs (Fig. 2A). Furthermore, both hPolλ forms were capable to excise a dRP residue, generated by the 5′ cleavage of an AP site by human apurinic/apyrimidinic endonuclease (hAPE) (Fig. 2B,C). The combination of polymerization and dRP lyase activities present in the W438 variant allows to complete BER *in vitro* as efficiently as in the case of the R438 wild-type hPolλ form (Fig. 2D). Moreover, there was no diference between the two isoforms in the capacity to insert either dCTP or dATP in front of a 8-oxoG lesion, or in its further extension (Figure S1). Therefore, we can conclude that substitution of arginine 438 to tryptophane in hPolλ, as it occurs in the natural W438 variant, does not significantly affect its *in vitro* catalytic efficiency.

### hPolλ W438 has a lower fidelity than hPolλ R438

A major property of DNA-dependent DNA polymerases is their DNA synthesis fidelity. It has been shown that a decrease in the fidelity of a particular DNA polymerase can lead to detrimental cellular effects, including cell death, cancer, and other genetic diseases [25], [26]. Moreover, several examples exist of point mutations in Polβ causing a dramatic decrease in fidelity [27]–[32]. To determine whether the tryptophan substitution at position 438 affects hPolλ fidelity, we measured base substitution and insertion-deletion error rates by the wild-type (R438) and the W438 hPolλ during a short gap filling reactions in M13mp2 reversion assays [27], [28]. Base substitution errors that revert a TGA codon encoding a faint blue plaque phenotype are scored as dark blue revertants. The DNA products yielded mutant frequencies of 9.0±4.4×10^−4^ (hPolλ R438) and 31±12×10^−4^ (hPolλ W438), as an average of four independent determinations (p = 0.0007). Sequence analysis of DNA amplified from blue-plaque revertants allows error rates to be calculated for each hPolλ form. The results (Table 1) indicate that, in comparison to the wild-type, the W438 variant is less accurate for T to C and G to T substitutions by factors of 4-fold (p = 0.001) and 8-fold (p<0.022), respectively. In contrast, the insertion-deletion mutant frequencies of the two forms were similar (data not shown) when examined using a 6-nucleotide gap-filling substrate containing a template TTTT run in the +1 reading frame.

**Table 1:** Base substitution specificity of allelic variant W438 of human Polλ during short gap filling synthesis.

Enzyme	Mispair	# mutants^c^	E.R.^d^×10^−4^
**Polλ R438** a	T • dGTP	6	**2.5**
	T • dCTP	0	≤0.4
	T • dTTP	0	≤0.4
	G • dATP	0	≤0.4
	G • dGTP	0	≤0.4
	A • dCTP	31	**13**
	A • dGTP	1	0.4
	A • dATP	1	0.4
**Polλ W438** b	T • dGTP	26	**10**
	T • dCTP	0	≤0.4
	T • dTTP	0	≤0.4
	G • dATP	9	**3.5**
	G • dGTP	0	≤0.4
	A • dCTP	57	**22**
	A • dGTP	3	1.2
	A • dATP	0	≤0.4

a Mutation Frequency (Polλ R438): 9.8×10^−4^.

b Mutation Frequency (Polλ W438): 22.05×10^−4^.

c Total sequenced: Polλ R438->42; Polλ W438->96.

d The error rates are calculated based on sequencing mutants from two different reactions with each enzyme, and using the average mutant frequency for those two experiments, i.e., 9.8×10^−4^ and 22.05×10^−4^ for Polλ R438 and Polλ W438, respectively.

**Figure 3:**
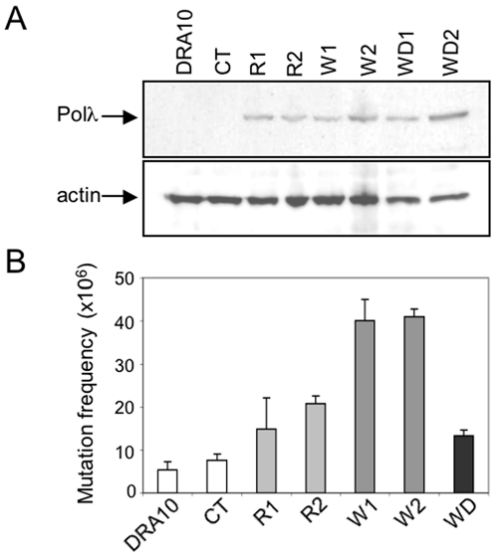
The W438 variant of hPolλ increases mutagenesis *in vivo*. (A) Immunoblotting of hPolλ in the cell lines overexpressing hPolλ R438 (R1 and R2) and W438 (W1, W2 and the catalytic inactive WD) as compared to control CHO-Dra10 and CT cells (cells transfected with the empty expression vector). As the endogenous levels of hamster Polλ were not detectable, normalization was carried out with actin antibodies. (B) Mutation frequency of hPolλ-overexpressing clones. To determine spontaneous mutagenesis, cells were plated at a density of 10^6^ cells and exposed to 20 µM 6TG-containing media in order to count the number of HPRT mutants. After 1 additional week, macroscopic colonies were scored and mutation frequencies were calculated by correcting for plating efficiency. The data presented is the average of three independent experiments. The p values relative to the control CT are 0.019, 4.6 10^−3^, 3.1 10^−3^, 1.3 10^−4^, and 0.045, for R1, R2, W1, W2 and WD, respectively.

We next investigated whether overexpression of the hPolλ W438 variant could affect mutation frequency *in vivo*. Thus, an eukaryotic expression vector harbouring the W438 variant or the R438 wild-type form of hPolλ was transfected into Chinese hamster ovary (CHO) cells to obtain isogenic strains expressing each isoform (R1 and R2, for R438; W1 and W2, for the W438 variant). Immunoblotting of cellular extracts using murine Polλ polyclonal antibodies [4] confirmed similar hPolλ levels in all overexpressing cell lines (Fig. 3A). The poor detection of the endogenous hamster Polλ in the control strains Dra10 and CT (transfected with the empty vector) precluded a proper quantification of the absolute ectopic expression levels. We next measured the frequency of spontaneous mutations by the conventional hypoxanthine phosphoribosyl transferase (HPRT) methodology that tests the appearance of a mutational event leading to 6-thioguanine resistance (6-TG^R^) [33]. Figure 3B shows that the mutation frequency corresponding to clones R1 and R2 was 2- to 3- fold higher than in control cell lines (clones DRA10 and CT). This agrees with previous data showing that Polβ overproduction increases (up to 4-fold) mutagenesis using the same assay [33]. More interestingly, overexpression of the W438 variant (clones W1 and W2) produced up to an 8-fold increase in the *in vivo* mutation frequency (Fig. 3B). Importantly, expression of a polymerization-deficient W438 variant (clone WD; see also Materials and Methods) did not produce such a mutation increase (Fig. 3B). Overall, these results show that cells expressing the W438 variant form of hPolλ, shown to be error-prone *in vitro*, acquire increased mutability.

### 3D-structure of the W438 hPolλ variant

The structure of hPolλ in complex with DNA and an incoming dNTP has recently been reported [34]. This structure indicates that R438 is a surface residue located in a loop of the palm subdomain (Figure S2). The B-factors in this loop are high, suggesting a certain degree of disorder and, in fact, the density for R438 was not sufficient to build the entire side chain. To try to understand the effects of the R438W substitution we crystallized the 39 kDa domain of the W438 variant under the same conditions already reported for the R438. We were able to obtain crystals that diffracted to low (2.8 Å) resolution. The density was of sufficient quality to assess that the overall fold of the W438 variant is identical to that of the R438 wild-type form, indicating that the R438W substitution does not result in a major conformational alteration (the rmsd between the R and W structures is 0.509 for 324 C-a atoms). However, the loop containing residue 438 had a high degree of disorder and no density was observed for the W438 side chain (not shown), suggesting that the R438W substitution might result in local structural alterations. It can be speculated that such a flexibility could be relatable to a necessary conformational change of this loop (that could be altered by the W438 polymorphism), required to form the enzyme∶DNA∶dNTP ternary complex and thus having an impact on polymerization fidelity. Interestingly, this loop is located next to the N-terminal end of α-helix M in hPolλ, and it is known that a mutation in this α-helix in Polβ can severely affect enzyme fidelity [35]. Amino acid sequence comparison of DNA polymerases lambda from different species indicates that an arginine residue is not highly conserved, being substituted for some other amino acid residues as, lysine, glutamine, and even alanine. As a tryptophane is not included among the wild-type options, we would favour that the observed effects are probably due to that particular bulky aromatic amino acid (tryptophane) substitution of the human Polλ W438 variant.

### Expression of the W438 hPolλ variant reduces cellular NHEJ activity

**Figure 4:**
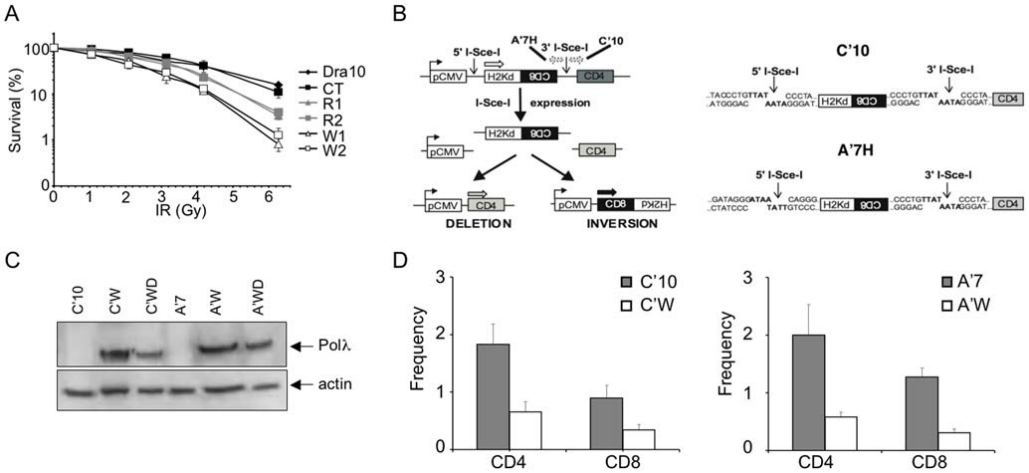
Defective NHEJ in cells expressing the polymorphic W438 hPolλ variant. (A) IR sensitivity of the DRA10 cell lines expressing the different forms of hPolλ described in Figure 3. (B) Substrate used to measure NHEJ (left panel) and representation of the sequences of the I-*Sce*-I restriction sites in the C′10 and A′7 cell lines (right panel). (C) Expression of the different forms (active: C′W, A′W; inactive: C′WD, A′WD) of W438 hPolλ in C′10 and A′7 analyzed by Western blot. (D) Evaluation of the deletion (CD4) and inversion (CD8) events in C′10 (left panel) and A′7 cells (right panel) expressing the empty vector (white bars) and the indicated W438 variant of hPolλ (grey bars). Results are the mean+/−SD of 3 independent experiments, *represent significant statistical difference (P<0.05), **represent significant statistical difference (P<0.005).

Considering the proposed role of Polλ in DSB repair, we examined survival after ionizing radiation (IR) exposure of the different hamster cell lines described above (Fig. 3), expressing comparable levels of either R438 or W438 forms of hPolλ. Expression of the W438 variant conferred a significantly higher sensitivity to IR relative to control or R438 expressing cells (Fig. 4A). Identical results were obtained in transfected human MRC5 fibroblasts (Figure S3, partA). Interestingly, this HPRT hypersensitivity, induced by the presence of the W438 form, was not observed in a NHEJ-defective cell line (XRCC4^KD^cells) (Figure S3, partB), suggesting that the presence of the W438 hPolλ variant may result in a defective NHEJ. We therefore evaluated the effect of both hPolλ isoforms directly on the cellular NHEJ activity. Cellular models (C′10 and A′7 cell lines) with stably integrated NHEJ substrates [20, 36; Fig. 4B] were transfected by each isoform (R438 and W438 variant) to obtain cellular clones (C′R, C′W, A′R, A′W), expressing similar levels as confirmed by immunoblotting (Fig. 4C). The fragment generated after I-Sce-I transfection allows to measure deletion and inversion events. In the first cell line (C′10) the two I-Sce-I sites are in direct orientation resulting in CD4 expression after deletion events (the most frequent events) or CD8 expression after invertion events. Expression of the WT form of hPolλ (R438) did not affect the generation of deletion (CD4) and inversion (CD8) events relative to control cells as previously reported [20]. In contrast, expression of the W438 form (C′W clone) decreased by 2.5-fold the CD8 events (inversion) and by 3-fold the frequency of CD4 events (deletion) relative to control cells (Fig. 4D-left panel). This was confirmed by the use of an additional cell line (A′7) in which the two I-Sce-I sites are in inverted orientation (Fig. 4B) resulting in CD4 expression after deletion events (still the most frequent events) and CD8 expression after inversion events. Again, while no effect was shown with the WT form as previously observed [20], cells expressing the R438W variant (A′W) produced a significant decrease in the generation of deletion events (CD4) and inversion events (CD8) (4- and 3-fold decrease respectively relative to control cells) (Fig. 4D-right panel). These data demonstrate that expression of the W438 polymorphic variant of hPolλ decreases the cellular NHEJ activity, probably interfering with the maturation of both non-complementary and, at a lesser extend, complementary DNA ends during the repair of DSB generated by I-Sce-1.

### Expression of the W438 hPolλ variant leads to chromosomal aberrations

**Figure 5:**
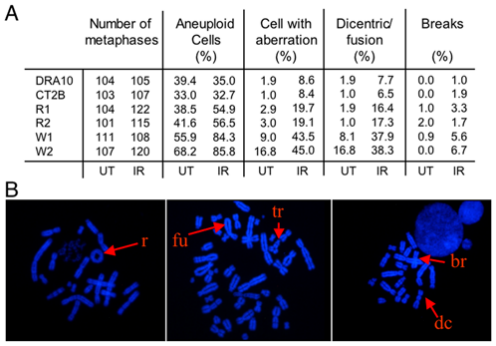
Karyotype analysis of W438 hPolλ-expressing cells. (A) Increased chromosomal instability in W438 hPolλ-expressing cells. Metaphase spreads of either untreated (UT) or 2 Gy-treated (IR) cells corresponding to either control clones (DRA10 and CT2B), or to clones overexpressing either R438 (R1, R2) or the W438 variant (W1, W2) of hPolλ. (B) Examples of chromosomal aberrations observed in cells expressing the W438 variant of hPolλ.

Since defects in the NHEJ pathway are known to lead to accumulation of chromosomal aberrations either spontaneously or after IR treatment [37], [38], we performed karyotypic analyses of the different cell lines described above. Examination of metaphase spreads showed a much higher generation of spontaneous or IR-induced chromosomal aberrations in cells expressing the W438 polymorphic variant of hPolλ relative to control cells or cells expressing the R438 hPolλ (Fig. 5). Dicentric and end-to-end chromatid fusion, hallmarks of a defective NHEJ, were the most common abnormalities seen in the W438 hPolλ expressing cells. We also found rings, triradial structures, and chromatid breaks (examples of some of these aberrations are given in Fig. 5). The level of spontaneous or IR-induced aneuploidy was also significantly enhanced in the W438 hPolλ expressing cells relative to control cells or cells expressing WT (R438) hPolλ. After IR, the effect is dramatic as aneuploidy reached 85% in the case of cells expressing the W438 variant (Fig. 5). Overall, these results show that expression of the W438 hPolλ induces major chromosomal instabilities, spontaneously or after IR treatment.

### Requirement of a functional DNA polymerase activity for the W438-associated phenotypes

**Figure 6:**
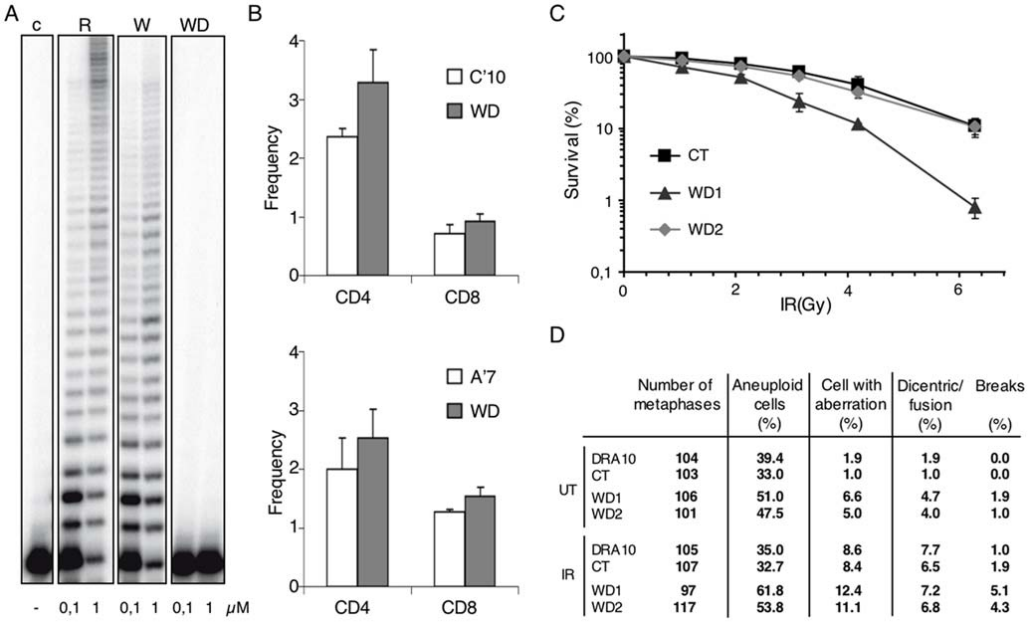
Requirement of a functional DNA polymerase activity for the W438-associated phenotypes. (A) DNA polymerization activity of the mutant form (WD) of the hPolλ W438 was negligible in comparison to that of the natural variants R438 and W438. All the phenotypes associated with expression of the W438 variant into DRA10 cells (analyzed as described in Materials and Methods) were abolished when the inactive WD mutant was expressed. Thus, mutant WD displayed a normal NHEJ activity (B), a normal sensitivity to IR (C), and a normal karyotype analysis (D).

A cDNA coding for a catalytically inactive form of hPolλ, in which two catalytic aspartates (D427 and D429) were changed to Ala, was obtained by site-directed mutagenesis on the pRSETB plasmid carrying the cDNA sequence of the variant W438 hPolλ gene. This construct was used to overproduce the inactive (dead) variant W438 (WD) in *E. coli*, that was purified as previously described [8]. As expected, DNA polymerization activity in the mutant form (WD) of the hPolλ W438 was negligible (Fig. 6A). The corresponding cDNA was transferred to the pIRES vector, and transfected into DRA10 cells, as described in Materials and Methods, to obtain the “dead” mutant clone WD. As shown in Figure 6, all the phenotypes associated with expression of the W438 variant were abolished when the inactive WD mutant was expressed. Thus, mutant WD displayed a normal NHEJ activity (Fig. 6B), a normal sensitivity to IR (Fig. 6C), and a normal karyotype analysis (Fig. 6D).

That indicates that the observed phenotyes intrinsically depend on the polymerization capacity of the variant DNA polymerase, but not indirectly due to any DNA repair disbalance produced by eventual titration (via protein∶protein interactions) of NHEJ factors by the overproduced polymerase. Therefore, it must be assumed that the more frequent errors made by the polymerase variant are the direct responsible for the observed phenotypes.

## Discussion

We describe here a coding SNP in exon 8 of the *POLL* gene resulting in an amino acid substitution at position 438, coding for a tryptophan (W) instead of an arginine (R). This polymorphism did not affect dramatically the protein structure at the polymerization active site and consistent with this observation, *in vitro* assays using purified hPolλ forms R438 and W438 did not show significant differences neither in polymerase and dRP lyase activities, nor in reconstituted BER reactions. However, we found that purified W438 hPolλ displays higher error rate *in vitro*, and that its expression in mammalian cells severely affects maintenance of the genome stability by enhancing mutation frequency, by compromising NHEJ, and by generating spontaneous or IR-induced chromosomal aberrations. We also prove that the observed defects are directly linked to the activity of the polymerase ruling out the hypothesis of any effect of imbalance in protein/protein interactions. How does the reduced fidelity of the W438 variant could affect NHEJ and chromosome instability? Whereas the overall effects observed on NHEJ efficiency and chromosomal instability are very dramatic, it is not obvious that they might be simply due to the observed/quantified effect (3-8 fold) on nucleotide insertion fidelity. However, it is important to emphasize that although the fidelity measurements are given as a mean value, figures can be higher when considering particular mismatches, and they can be even much higher when considering mismatches made in a particular sequence context (hot spots). On the other hand, reduced polymerization accuracy may have a more drastic effect during NHEJ, as the errors introduced during such unstable gap-filling synthesis could hinder ligation, the final step of the repair pathway. Alternatively, we cannot rule out the possibility that the W438 mutation affects the direct interaction of this variant Pol lambda with some other NHEJ factors.

Interestingly, restriction fragment length polymorphism (RFLP) analysis of a collection of above 300 cases-control indicated that the W438 variant is associated to rectal cancer, and the allelic frequency of the W438 allele is 0.27 in the caucasian population (manuscript in preparation). The findings shown here demonstrate that overexpression of a cancer-related natural variant of one specialized DNA polymerase can be associated to genetic instability at the chromosomal level, probably due to a defective NHEJ. There is evidence that overexpression of Pol lambda, as well as Pol beta, occurs frequently in human tumors [39]. Analysis of the heterozygote population R/W in colorectal cancer patients shows that both alleles are expressed at the same level (our unpublished data). Therefore, expression of the W438 allele does not likely affect the global DSB repair capacity in these heterozygots in a normal physiological situation, but may predispose the cells, perhaps due to DNA repair fidelity issues, to accumulate chromosomal aberrations when excessive DSBs are generated, as it occurs in pre-cancerous hyperplasia tissues [40], or when an additional factor involved in DSB repair is altered.

In spite of their key role in DNA repair, polymorphic forms of DNA repair polymerases have been poorly described, except in several studies carried out with Polι and Polβ, where a significant number of genetic alterations were reported [35], [21]–[23], [41], [42]. More recently, an association between the presence of altered forms of Polβ and their overall DNA repair capacity, resulting in cellular transformation, has been described [24], [32]. Identification of which mutations in human genes are determining the genetic basis of diseases is a challenge. In this sense, the vast data generated during the human genome project only provide a minimal help to establish the relationship between sequence variation and susceptibility to disease. In addition to polymorphisms that are simply associated to causative mutations, those responsible for functional differences that directly contribute to disease are of greater importance. Loss of proofreading by a replicative polymerase as Polδ increases spontaneous tumour development in mice [43]. Is the human Polλ mutation W438 actually causative or contributory to oncogenesis? Although we do have shown an association of this mutation with rectal cancer (manuscript in preparation), it is unlikely that this sole mutation is causative to oncogenesis, but it could contribute to enhance genetic instability, one of the major hallmark in cancer cells. As proposed by Hanahan and Weinberg [44], a variant DNA repair enzyme, as that described here, might accelerate carcinogenesis by increasing genetic instability, at the nucleotide as well as at the chromosomal levels, which in turn confers a selective growth advantage during cancer-cell evolution.

## Materials and Methods

### PCR-SSCP, cloning and sequencing

A panel of cDNAs from normal and tumoral human tissues (Clontech) was used as a template to search for possible genetic alterations in the hPolλ gene. The set of overlapping primers selected was used to cover the entire coding sequence of the gene. The sequences of primers used were: hPolλex1s:5′TAGCTTGGCCAGTAGTCGACC; hPolλex3as:5′GAAGGGAGCTCAGCCACTC; hPolλex2s:5′ATACTTCAATGGATCCCAGGG; hPolλex4as:5′TTGGGTGTTTGGTGCCTC; hPolλex4s:5′GAGGCACCAAACACCCAA; hPolλex5as:5′TCCACTTGTCTCCCTGAACAC; hPolλex5s:5′TCCATAAGCCTGTCACCTCG; hPolλex7as: 5′ GGGCATACGTTCCAGGAAG. cDNA templates were 5-fold diluted and the resulting aliquot was used as 5x stock for PCR. All PCR reactions (10 µl) were conducted using 1 µM of each primer, 0.25 mM dNTPs, 1x Taq buffer (10 mM Tris pH 8.5, 50 mM NaCl, 1,5 mM MgCl_2_, gelatin, 0.2 mg/ml bovine serum albumin (BSA)) and 0.25 U of Taq polymerase. Cycle parameters were as follows: 94°C, 15 s; 62°C, 30 s; 72°C, 15 s; 35 cycles. PCR products were then subjected to SSCP analysis carried out as described [45], [46]. Fragments exhibiting altered mobility patterns were cloned into the TA cloning vector pCRII (Invitrogen) and sequenced on an ABI 373 (Applied Biosystems) automatic sequencer.

### Enzymes, DNA substrates and nucleotides

Synthetic oligonucleotides purified by polyacrylamide gel electrophoresis (PAGE) were obtained from Life Technologies. Ultrapure deoxynucleoside triphosphates, activated calf thymus DNA, [γ-^32^P]ATP, [α-^32^P]dATP, [α-^32^P]dCTP and [α-^32^P]ddATP (3000 Ci/mmol) were from Amersham Pharmacia Biotech. Terminal deoxynucleotidiltransferase (TdT) was from Promega. Human recombinant Polλ (R438 and W438 forms) were overexpressed in *E. coli* and purified as described [8]. Human uracil DNA glycosilase (hUDG) and hAPE were generous gifts of Dr. Samuel H. Wilson (NIEHS, NC). T4 polynucleotide kinase (PNK) was from New England Biolabs.

### DNA polymerization assays

Reactions on activated DNA were carried out as described in [8] and were initiated by adding either the indicated or a fixed amount (60 ng; 35 nM) of each hPolλ variant, in the presence of different concentrations of the indicated dNTPs. Polymerization activity, determined as total dNMP incorporated, was calculated from the amount of radioactivity present in the excluded volume, determined by counting Cerenkov radiation. Primer extension assays were performed as described in [47] with 100 nM of either hPolλ R or hPolλ W and the indicated concentration of dNTP and incubated at 37°C for 20 min.

### dRPlyase activity and *in vitro* reconstitution of BER

Reactions were performed as previously described [9]. The reaction was initiated by adding different amounts of R438 or W438 forms and incubated for 20 min at 37°C.

### Fidelity assays

The base substitution reversion assay was performed as described [27]. Gap-filling reaction mixtures (20 µl) contained 50 mM Tris-HCl, pH 7.5, 10 mM MgCl_2_, 1 mM dithiothreitol, 2 µg of BSA, 4% glycerol, 1.6 nM gapped DNA, 500 µM each of dATP, dGTP, dCTP, and dTTP, 400 units of T4 DNA ligase, and 100 nM hPolλ (either R438 or W438 forms). After 1 h incubation at 37°C, the products were separated on an agarose gel, and the covalently closed circular DNA products were electroeluted from gel slices. DNA products were introduced into *E. coli* by electroporation and followed by plating as described [48].

### Generation of hPolλ overexpressing cells

hPolλ expressing plasmids pIRES-hPolλ R438 and pIRES-hPolλ W438 were constructed by PCR amplification from a pRSETB plasmid carrying the cDNA sequences of either the wild-type hPolλ gene (R438 form) or the variant W438 hPolλ gene, respectively, and cloning into pIRES vector (Clontech). The construction of a catalyticaly inactive mutant of W438 (WD) was carried out as described [20]. Upon transfection in CHO-DRA10, XR-1 (XRCC4 mutant cells), and MRC5 cell lines, different clones (DRA10-R1; DRA10-R2; DRA10-W1; DRA10-W2; MRC5-R; MRC5-W; XR-1-W1; XR-1-W2) were obtained. Expression of the different hPolλ protein variants was measured by immunoblotting of total cellular extracts (75 µg) with Polλ polyclonal antibodies [4] and the actin antibodies AC-40 (Sigma-Aldrich Chemical Co).

### Mutagenesis assay in mammalian cells

For determination of spontaneous mutagenesis, replica cultures of cells were plated at the density of 5×10^5^ cells by plate (5 plates per experiment) and exposed to 20 µM of 6-thioguanine (6-TG) containing media in order to determine the number of HPRT mutants that are resistant to 6-TG treatment. After 8 days, plates were stained and macroscopic colonies of more than 50 cells were scored and mutation frequencies were calculated by correcting for plating efficiency.

### Cytotoxicity studies

Cytotoxicity of ionizing radiation was determined by clonogenic assay [20]. Survival was expressed as the plating efficiency of treated cells relative to the untreated control cells. Results are the mean+/−SD of 3 independent experiments.

### Measurement of cellular NHEJ activity

NHEJ activity was measured as described in [20]. The C′10 and A′7 cell lines were cultured in DMEM medium (GIBCO BRL, France) as previously described [34]. The C′W and A′7W clones, and the C′WD and A′7WD clones were obtained after transfection with the pIRESpuro2 vector (Clontech) containing the cDNA coding for the W438 hPolλ (W) or the inactive form of the W438 hPolλ (WD), respectively. Individual clones were obtained after transfection with jetPEI (Qbiogen, Illkirch, France) and selection with puromycin (5 µg/mL).

### Karyotype analyses

Cells were unirradiated or irradiated at 2 Gy as described previously. Karyotype analyses were performed as described in [49]. Chromosomal distributions and aberration percentages included the analysis of at least 100 metaphases spreads for each experiment.

## Supporting Information

Figure S1 TLS of 8oxoG lesions by hPolλ variants R438 and W438.The scheme shows the sequence of the DNA used to analyze nucleotide insertion reactions opposite dG or 8oxoG (A) or extension of primers paired to 8oxoG (B). Reactions were carried out as described under Materials and Methods, with 100 nM of either R438 or W438 isoforms of hPolλ, and using 1 µM of each indicated dNTP (A) or the indicated concentrations of dG (B). Extension of the 5′ end labeled primer (*) was examined by PAGE.
(4.91 MB EPS)

Figure S2 Location of the R438 residue in the crystal structure of hPolλ. Ribbon representation of the ternary pre-catalytic complex of hPolλ (PDBid 1XSN). Arg438 (magenta; pointed with an arrow) is located in a loop (shown in green) in the palm subdomain. This loop is next to the N-terminal end of α-helix M. Both α-helices M and N (shown in dark gray) are critical to position the substrates and assemble the nascent base pair binding pocket. The DNA duplex is shown in light blue. The templating base is yellow and the incoming ddTTP is red.
(5.26 MB EPS)

Figure S3 Cell survival after ionizing radiation. Cell survival after ionizing radiation for MRC5 control cells (MRC5), or expressing the R438 (MRC5-R) or W438 (MRC5-W) forms of hPolλ (A), and for NHEJ-defective cells (XRCC4-defficient), control (XR-1), complemented by XRCC4 (X4-V; 20), or expressing the W438 (XR-1-W1; XR-1-W2) form of hPolλ (B).
(4.79 MB EPS)
