# Correction: Characterization of a Natural Mutator Variant of Human DNA Polymerase λ which Promotes Chromosomal Instability by Compromising NHEJ

**DOI:** 10.1371/annotation/c20f5062-ddb8-4bc2-b374-f9a901a23170

**Published:** 2009-10-09

**Authors:** Gloria Terrados, Jean-Pascal Capp, Yvan Canitrot, Miguel García-Díaz, Katarzyna Bebenek, Tomas Kirchhoff, Alberto Villanueva, François Boudsocq, Valérie Bergoglio, Christophe Cazaux, Thomas A. Kunkel, Jean-Sébastien Hoffmann, Luis Blanco

The equal contributorship of authors 1 and 2, Gloria Terrados and Jean-Pascal Capp, was erroneously omitted. Gloria Terrados and Jean-Pascal Capp should be listed as having contributed equally to this work.

